# The role of qualitative research in adding value to a randomised controlled trial: lessons from a pilot study of a guided e-learning intervention for managers to improve employee wellbeing and reduce sickness absence

**DOI:** 10.1186/s13063-016-1497-8

**Published:** 2016-08-09

**Authors:** Jill Russell, Lee Berney, Stephen Stansfeld, Doris Lanz, Sally Kerry, Tarani Chandola, Kamaldeep Bhui

**Affiliations:** 1 Centre for Primary Care and Public Health, Barts and The London School of Medicine and Dentistry, Queen Mary University of London, London, UK; 2 Centre for Psychiatry, Wolfson Institute of Preventive Medicine, Barts and The London School of Medicine and Dentistry, Queen Mary University of London, London, UK; 3 School of Social Sciences, University of Manchester, Manchester, UK

**Keywords:** Mixed methods, Qualitative research, Workplace stress

## Abstract

**Background:**

Despite the growing popularity of mixed-methods studies and considerable emphasis on the potential value of qualitative research to the trial endeavour, there remains a dearth of published studies reporting on actual contribution. This paper presents a critically reflective account of our experience of the actual value of undertaking qualitative research alongside a pilot cluster randomised controlled trial of a guided e-learning intervention for managers in an NHS Mental Health Trust to improve employee wellbeing and reduce sickness absence.

For the qualitative study we undertook 36 in-depth interviews with key informants, managers and employees. We observed and took in-depth field notes of 10 meetings involving managers and employees at the Trust, and the two qualitative researchers acted as participant observers at steering committee and monthly research team meetings. We adopted a narrative methodological orientation alongside a thematic approach to data analysis, eliciting a rich account of the complexities of managing stress at work.

**Results:**

We identified two key overarching roles played by the qualitative research: ‘problematising’ and ‘contextualising’. Specifically, the qualitative data revealed and challenged assumptions embedded in the trial about the nature of the learning process, and exposed the slippery and contested nature of abstracted variables, on which a trial depends. The qualitative data challenged the trial’s logic model, and provided a rich understanding of the context within which the trial and intervention took place.

**Conclusions:**

While acknowledging the ever-present tension in mixed-methods research between the requirements of quantitative research to represent the social world as abstracted variables, and the goal of qualitative research to explore and document the complexity of social phenomena, we adopted a pragmatic position that enabled us to engage with this tension in a productive and partially integrative way. Our critically reflective account of the praxis of integration illuminated opportunities and challenges for maximising the value of qualitative research to a trial. This paper sets out tangible illustrative lessons for other mixed-methods researchers endeavouring to get the most from qualitative research.

**Trial registration:**

This study is registered as ISRCTN58661009. Registration was submitted on 22 April 2013 and completed on 17 June 2013.

## Background

In 2014, the National Institute for Health Research (NIHR) Health Technology Assessment (HTA) programme published an in-depth research report on maximising the value of combining qualitative research and randomised controlled trials (RCTs) in health research [1]. The study found that far more is known about the *potential* value of qualitative research to trials than the *actual* value, primarily because published accounts of RCTs fail to report details of how, *in practice*, qualitative research contributed to and impacted on the trial. More commonly, researchers report on the findings and conclusions of the qualitative research alongside the trial findings and its independent contribution to knowledge, rather than explicitly engaging with the question of how qualitative research *enhances* the trial [2].

Researchers have identified a range of potential benefits of qualitative research to the trial endeavour [2–6], including facilitating interpretation of trial findings, exploring stakeholder perceptions of the feasibility and acceptability of an intervention, facilitating understanding of the effect of context in which an intervention is delivered, and adopting a challenging role, for example, by problematising the trial’s underlying theory of change and logic model. If undertaken as part of a pilot or feasibility trial, qualitative research also has the potential to feed into the design of a full study.

This paper explores the role of qualitative research in adding value to an RCT, through a case study of our experience as a mixed-methods research team engaged in a pilot study of a guided e-learning intervention for managers in the workplace to improve employee wellbeing and reduce sickness absence, referred to as the ‘GEM’ study (Guided E-learning for Managers). A recent paper in this journal reported on an interview study of researchers with experience of undertaking qualitative research alongside RCTs of health interventions. The authors recommended that: *‘researchers, funding agencies, universities, and journal editors place more value on articulating the impact of qualitative research undertaken with trials in order to reap the considerable benefits of this endeavor’* [7]. The aim of this paper is to articulate our practical experience, and offer tangible examples and methodological lessons to other mixed-methods research teams wanting to maximise the impact of qualitative research on the trial endeavour. Interview studies, such as that reported by O’Cathain and colleagues, offer important general information on the views and experiences of mixed-methods researchers, and provide the backdrop to this paper. However, our aim is that by locating our critically reflective account of the opportunities and challenges for maximising value from qualitative research within a case study of a specific trial, we provide additional illumination on the actual practicalities of undertaking mixed-methods research.

We begin by summarising the trial and intervention, and the key findings to emerge from the trial, and present details of the qualitative component of the GEM study. We identify and illustrate two key overarching sense-making roles played by the qualitative research: ‘problematising’ and ‘contextualising’. Specifically, we explore how the qualitative data questioned assumptions embedded in the trial about the nature of the learning process; exposed the ‘slippery’ and contested nature of abstracted variables on which an RCT depends [8]; challenged aspects of the trial’s logic model, and provided a rich understanding of the context within which the trial and intervention took place.

In the final section of the paper we unpack the concept of ‘integration’. O’Cathain and colleagues suggest that a key condition for maximising value in mixed-methods studies is *integration* of quantitative and qualitative components [1], so that researchers achieve a *‘whole greater than the sum of the parts’* [9]. However, the review by Lewin et al. of trials which had associated qualitative work found that, for most cases, there was *‘no indication of integration of qualitative and quantitative findings at the level of either analysis or interpretation’* [4]. We explore what it means in practice to ‘integrate’ quantitative and qualitative research, in terms of both process and research findings.

This paper acknowledges the long-standing philosophical and methodological debates about the relationship between qualitative and quantitative research, and the question of the commensurability of the interpretivist and positivist paradigms within which these different research approaches are located [10–14]. Positivism places high value on experimentation and is characterised by formal hypotheses, careful measurement, and drawing of inferences about a phenomenon from a sample to a stated population. The positivist researcher assumes that understanding social phenomena is primarily a problem of objective measurement. Interpretivist researchers assume that social reality is produced and reproduced through the actions and interactions of people. Hence, social reality can never be objectively known or unproblematically studied; it can only be understood in context by exploring people’s interpretations of it [15]. Some argue that the ontological (nature of reality) and epistemological (nature of knowledge) assumptions of these research traditions are so fundamentally different that *‘accommodation between paradigms is impossible’* [16] (known as the ‘incompatibility thesis’ [17]). Others suggest that despite continued presence of the ‘paradigm wars’ in social research [14], the growth of mixed-methods research represents a *‘third research paradigm’* whose *‘time has come’* [17]. We aligned ourselves with the pragmatic position of mixed-methods research, *‘rejecting the either/or choices presented by the incompatibility thesis’* [12]. Pragmatism *‘endorses pluralism and carefully considered eclecticism (e.g. different, even conflicting theories and perspectives can be useful; observations, experience, and experiments are all useful ways to gain an understanding of people and the world)’* [12].

## The trial

A summary of the rationale, methodology and findings from this trial has been published elsewhere [18]. Briefly, the ‘GEM’ study addressed the public health priority of psychosocial work stress and mental health in the workplace. It took place in an NHS Mental Health Trust between June 2013 and April 2014, and was designed as a cluster RCT. Four hundred and twenty-four employees and 41 managers from four clusters within the Trust participated in the study; the intervention was applied in three clusters and one cluster acted as the control. The intervention comprised a guided e-learning programme based on the Health and Safety Executive management standards for the reduction of work-related stress [19, 20]. The e-learning programme consisted of six modules, covering the health, economic and legal issues of employee pressure at work, and how managers could help individuals and teams to reduce stress. Modules were expected to each take 10–30 minutes to study, with some modules including additional learning activities to encourage managers to apply their learning to their work situation. Managers were invited to a facilitated induction session at the beginning of the trial, and a second session to discuss progress during the course of the intervention. The intervention took place over a 3-month period.

For the employees participating in the trial, levels of wellbeing (assessed by the Warwick Edinburgh Mental Wellbeing Scale) and rates of sickness absence were measured approximately 3 months before and 3 months after the e-learning intervention. These constituted the primary outcome measures of the trial. The trial’s underlying theory of change was that through participation in and adherence to the e-learning programme, managers would improve their management competencies and change their behaviour towards employees. Change in managers’ behaviour would result in improved wellbeing and reduced stress among employees. Increased wellbeing would be related to employees taking less sickness absence.

The results of the pilot trial showed little positive effect on employee wellbeing and sickness absence. In fact, employee wellbeing decreased over the trial period, although it decreased marginally less in the intervention clusters than the control cluster. Sickness absence data showed no evidence of effect. In the subsequent sections of this paper we explore the sense-making role of qualitative research in relation to these findings.

## The qualitative study

The aims of the qualitative study as defined in the funded study proposal were to assess the views of key informants about the positive and negative aspects of the intervention; describe the context within which the intervention took place (organisational, local and national policy); explore the acceptability, ease of use and perceived usefulness of the intervention and managers’ experience of it; identify suggested modifications to the intervention; build a picture of how employees conceptualised and experienced stress and wellbeing at work, and their perceptions of their managers’ role in managing stress. Additionally, drawing on others’ accounts of how qualitative research can add value to a trial [4, 7], it was agreed by the research team that the qualitative study would aim to:

- Help draw out the intervention’s logic model/theory of change
- Document how the implementation process of the intervention and trial unfolded
- Explore whether the intervention was implemented as intended
- Explore effects of the intervention that were difficult to measure quantitatively
- Help with interpreting quantitative findings and understanding why particular aspects of the intervention did or did not work
- Offer case study illustrations to complement quantitative findings

## Methods

The qualitative component of the GEM study comprised in-depth interviews with key informants, managers and employees, ethnographic observation of a series of study meetings for managers and steering committee and study team meetings, and the collection of ‘free text’ data from the pre- and post-intervention questionnaires sent to employees as part of the trial. We adopted a narrative orientation to interviewing [21], meaning that interview questions encouraged respondents to recount stories of specific, anonymised cases and incidents, as a way of eliciting a rich and reflective account of the complexities of managing stress at work. A feature of narrative methods is their focus on concrete practices and instances, rather than, as is more typical of other interview methods, on abstract, generalised perspectives. We asked managers to talk through an instance of a specific case where an employee who they line managed had experienced stress, and how they had managed the particular case. We took a similar approach to asking questions about the managers’ experience of studying the guided e-learning intervention, and the same approach in interviews with employees to elicit employee stories of specific instances of stress at work.

We undertook 14 in-depth ‘key informant’ interviews with members of the study team and scientific steering committee, senior managers at the NHS Trust, and those involved with the development and implementation of the guided e-learning intervention. We followed up suggestions from these informants of additional key informants (for example, other researchers in the field of work stress and wellbeing). Interviews lasted between 30 minutes and 1 hour.

We adopted a purposive approach to sampling managers for interview to ensure a heterogeneous sample, including men and women from across the intervention and control group clusters. Twenty-one managers (out of the 41 in the intervention clusters who had consented to participate in the GEM study) were approached for interview and of these 11 agreed (the remaining 10 did not respond to the email invitation nor a reminder), giving a response rate of 52 % among managers in the intervention groups. Eight managers from the control cluster were invited to interview and two agreed (five did not respond to the email and one replied that she no longer held a managerial position). Thus, we undertook a total of 13 in-depth interviews with managers. Manager interviews took place between March and May 2014 (2 months or more after they had participated in the guided e-learning intervention), and lasted between 20 and 40 minutes. Interviews were audio-recorded, transcribed and anonymised.

We adopted a purposive approach to sampling employees for interview. Thirty-six employees from across the four clusters in the study were invited for interview (from the sample of 163 employees who had completed their follow-up questionnaires). Ten employees responded (a relatively low response rate of 28 %), and we were unable to arrange interviews with two of these employees, and subsequently undertook a total of eight employee interviews. Interviews took place in March and April 2014, lasted between 20 and 30 minutes, and were audio-recorded, transcribed and anonymised.

We observed and took in-depth field notes of 10 meetings at the Trust during the study period (an introductory meeting to introduce the study to managers, six facilitator-led induction and follow-up meetings for managers, a focus group discussion at which managers discussed preliminary findings from the interview phase, and two dissemination meetings at the end of the study for managers and employees). The two qualitative researchers acted as participant observers at the two steering committee meetings held during the course of the study, and at monthly study team meetings. Our dual role as participants in, and observers of, these meetings inevitably shaped the data we collected; nonetheless, we found this ‘auto-ethnographic’ data a helpful contribution to our sense-making of the unfolding of the research process.

Data analysis combined a thematic approach with a narrative orientation [22]. This meant that alongside an exploration of emerging themes, we paid attention to the story as a whole that respondents told, seeking to gain a situated understanding of their individual experiences and sense-making. We focussed primarily on content, on ‘what’ was being said, rather than the more in-depth structural (sequencing and language) or dialogical aspects of narrative analysis [23]. Our approach generated a rich picture of the subjective and situated experiences of workplace stress and the guided e-learning intervention.

Data analysis took place concurrently with data collection, enabling progressive focussing on emerging issues. The two qualitative researchers engaged in close readings of the transcripts of interviews and meetings, observational field notes, and associated documentation. We discussed our preliminary findings with members of the study team, individually and at team meetings, and with the steering committee, and drew on these discussions to scrutinise our data further and develop our analysis. At the end of the study we interviewed the principal investigator, asking questions to prompt reflection on the impact of the qualitative research on the trial; we asked similar questions through an email questionnaire to members of the steering committee. We draw on these findings in the discussion section of the paper.

The full findings of the study are reported in the project final report [18]. There we present an in-depth picture of the context in which the study took place, explore participants’ experiences of stress at work and the guided e-learning intervention, and their views about competencies and structures required for managing stress at work. In this paper we present a ‘meta-narrative’ of these findings, focussing on two overarching sense-making roles of qualitative research that emerged from the study: problematising and contextualising.

Arguably, a limitation of the findings presented here is that they are drawn from our experience as a mixed-methods research team involved in a single study, with its own specific characteristics and situated within a particular context. However, from a case study perspective, the aim of research is not to produce representative findings or ‘typical’ accounts, but rather to offer a rich account of the complexities and nuances of the particular, from which the reader can gain a deeper understanding of issues of interest [24]. The paradox of case study research is that through a focus on in-depth particularisation it aims to demonstrate how and in what ways findings may be relevant and transferable to other trial contexts, and in this sense, ‘generalisable’ [25].

We received ethical approval for the study from Queen Mary Research Ethics Committee (reference numbers QMREC2013/10 and QMREC1279). Informed consent was obtained from each participant in the study.

## Results

### The value of problematising

Problematising is a form of critical analysis fundamental to the qualitative research endeavour [26]. It involves exposing and questioning assumptions and presuppositions, scrutinising meanings, and challenging taken-for-granted notions. We present three illustrative examples of how the qualitative findings added value to the trial through the process of problematisation: the questioning of assumptions about the nature of the learning process, the measurement of adherence, and the notion of managerial competency.

### Questioning assumptions about the nature of the learning process

Although the guided e-learning intervention included some elements of experiential learning (for example, learning activities and group discussions which emphasised the role of active experience and reflection in shaping understanding [27]), the trial itself was predicated on a predominantly instructivist view of education, which depicts learning in terms of the accumulation of factual knowledge [28]. The trial assumed that learning could be assessed by the reproduction of factual knowledge: for example, learning gained from the intervention was measured simply by assessing pre- and post-intervention quiz scores.

By contrast, interviews and group discussions with managers emphasised the role of critical reflection in the learning process, and the value of interaction with other learners. For most managers the key value of the intervention was not the acquisition of new knowledge but the way in which it backed up existing knowledge, and encouraged reflection on managerial practice. In this sense, managers felt that it validated their own practice and thus helped build confidence:

I quite enjoyed the course because I didn’t really see things that were totally shocking to me or, ‘Oh! You should be doing that’. It reinforced that my way of doing it is alright, it’s acceptable … So I found that course sort of validated some of the stuff that I already do and sort of sends a message to me to carry on doing it that way’. (Manager, M4)

Those managers who attended the facilitated face-to-face sessions emphasised the role of learning through interaction with peers, and the collective sharing of experiences and concerns:

It was quite good to hear the other people in the room were having similar things, similar issues, similar thoughts, similar concerns. And it was good to express those concerns, I suppose, in a safe environment with no people higher up from myself looking down on you and judging you. So from that perspective, … it felt like a safe environment, just to discuss openly some of the issues that as managers we were concerned about and had raised. (Manager, M8)

Thus, the qualitative data highlighted the ways in which the intervention enabled participants to access domains of learning that were additional to knowledge acquisition, but these were unanticipated aspects of the learning process and not measured by the trial.

### Problematising the measurement of adherence

A key objective of the GEM pilot study was to investigate managers’ ‘adherence’ to the intervention – having agreed to participate in the trial, did managers ‘adhere’ to the educational intervention? Adherence was defined as managers having completed at least three of the six modules of the e-learning intervention. This was measured by the e-learning provider that hosted the e-learning programme recording the number of modules each participant completed. However, the qualitative interviews with managers indicated that ‘completing’ a module did not necessarily mean that managers had engaged with the learning activities that formed part of the educational programme. Indeed, several of the ‘adherent’ managers spoke of not having had time to complete the activities suggested in the e-learning programme, that arguably, as discussed above, comprised a critical part of the learning process. Furthermore, only a minority of managers attended the face-to-face facilitated sessions, again seen by the developers of the educational intervention as a critical part of the learning process.

This example highlights the tension between the demands of an experimental trial methodology, which requires that the social world is represented by abstracted variables (in this case a quantifiable measurement of adherence), and the social practices of those participating in the trial, which are unavoidably messier and more ambiguous than a variables-based research design is able to accommodate [29]. The qualitative findings thus raised important questions about the validity of the definition of ‘adherence’ adopted by the trial.

A further tension that emerged during the study was that, for the purposes of trial blinding, managers who received the intervention were instructed not to tell their employees that they were participating in the trial. The rationale behind this was to reduce the bias that it was thought could occur in employees’ responses to questions on wellbeing and their work environment if they knew that their managers were taking part in the intervention. Interviews with employees indicated that, apart from one or two exceptions, blinding had mostly worked, and employees were unaware of their manager’s involvement or not in the study. However, alongside this requirement for blinding, the e-learning materials included activities to prompt managers to discuss aspects of their learning with their employees, and to ask for feedback on their management style. One activity asked managers to conduct a ‘stress survey’ with their employees to identify and discuss problems and to work together to find solutions. Thus, a fundamental paradox emerged between the educational design of the intervention and the methodological requirements of the trial.

### Problematising the notion of competence

As identified above, the logic model of the trial was that through adherence to the educational intervention, managers would improve their managerial competencies as defined by the Health and Safety Executive’s Management Standards [30], and subsequently change their behaviour towards employees, which in turn would improve employee wellbeing and reduce sickness absence (although a limitation of the trial design recognised post hoc was that it did not include any measurement of changes in manager behaviour). A striking finding to emerge from the managers’ and employees’ accounts was the contrast between the description of required behavioural competencies suggested by the educational intervention (for example, the e-learning materials instructed managers to monitor employees’ workloads; help employees prioritise; develop rapport with employees; be fair with employees, and give constructive criticism) and the more nuanced, less tangible qualities that managers and employees identified when recounting specific instances of stress and managerial support.

In their accounts managers and employees emphasised accumulated experience, tacit knowledge, and virtues such as kindness and integrity, *‘being human’* and *‘compassionate’*. Interviewees’ accounts portrayed something more akin to practical wisdom [31] than competence, the latter construct being one which, it has been argued, tends to over-rationalise human and organisational behaviour, and reduces complex practices to a set of distinct behaviours and technical skills [32]. The narrative accounts, as illustrated in Table 1, generated a vivid and nuanced account of particular actors and events in context, and in this way highlighted the contingent, situated nature of managing stress, challenging the literal and linear rationality [33] underpinning the logic model of the trial. In the first narrative in Table 1, the manager makes subtle judgements about how best to juggle individual and organisational needs, and highlights the role of personal beliefs and values in deciding how to act. In the second example, the manager talks about *‘thinking outside the box’* and refers to the critical attribute of empathising.

**Table 1:** Managers’ stories of helping employees manage stress at work

It’s more of a personal nature for this member of staff. She’s going through a very difficult break up of a marriage, got young children too … it’s all blown up and all … really struggling, really having difficulties with it. I’m going out to see her on a fairly regular basis – I’ve been out to see her today, actually. Going out, giving all the support I can, refer her to occupational health, refer her to staff support. It’s a really difficult one because I’m sitting there saying, ‘Yes, yes. I hear that you’re not ready to come back. Yes, I hear what you’re saying to me,’ but on the other side of that is the fact that there’s a service need. She had a caseload of patients that we’ve had to share out with other people now, not everybody wants to go to another therapist. Therapy’s quite individualised and quite thought-provoking, and you’re sharing your soul to the devil, so to speak, aren’t you? That’s how it feels. So that’s difficult because it’s that balance of I hear what you’re saying, you’re in a really horrible place, I can’t imagine anything worse for you, but on the other side of that, I’ve got to get you back into work somehow… I think I’ve had to draw on compassion. I think I’ve had to draw on knowing the policy, knowing what I can and cannot allow her to do. The return to work policy, the phased return, all of that, I’ve had to look on that. I think I’ve had to draw my own personal beliefs and my own personal values, really, and be able to stand up and say, ‘I hear what you’re asking as a Trust. I hear what you’re saying as a Trust but I’m the person that’s in there, I’m the person that’s dealing with this individual, you know, I’ll bring her in to fail and that’s how I feel at the moment. I think she’s too fragile, too vulnerable to come back in at this precise moment but I’m also aware that if I take that to a more senior manager they may say I hear what you’re saying but she needs to get back in. (Manager, M6) I’ve got a member of staff who is still with me, who, last year was for some time, has been the main carer for her father, and whose become increasingly frail. Last year he was diagnosed with cancer, and was only given a couple of months to live. He subsequently lasted several more months than that. But, obviously, this caused her additional stress because it wasn’t only her work she had to juggle. She had to sort out carers to go in when she was in work, and also when she wasn’t in work … So she had a lot of stress around that … She also had a sister with learning disabilities who, although didn’t live with them, was sort of there in the background, and I think a lot of it was she was thinking how was she going to support her if anything happened to the father. So there were lots of stresses around that. I think (managing stress) for her it was very much about thinking outside the box [manager describes how she introduced flexible working hours for this employee]. What is it that I could do as a manager to support her as much as I could, to prevent her going off sick, because she would’ve gone off sick… And we were able to prevent that. And I suppose a lot of it for me was being able to empathise with her; having gone through bereavement of a close family member myself. You can think what would’ve been good for me at that time. (Manager, M2)	

### The value of contextualising

An increasingly acknowledged benefit of undertaking qualitative research alongside trials is its contribution to an understanding of the *context* in which a trial takes place [1]. This becomes even more important in a cluster RCT where the intervention is directed at the cluster (manager) level and the outcomes measured at the level of individual members (employees).

### Organisational, local and national policy pressures

In our study the qualitative research served to highlight the pervasive effects of the particular organisational and broader policy context within which the trial occurred. The study took place at a time of considerable change and uncertainty both within and beyond the NHS. Respondents cited pressures on NHS funding, restrictions on housing and other state benefits, and the impact of the Francis Inquiry [34] into failings in NHS care, as all affecting overall staff morale and wellbeing, and having, in the words of one respondent, *‘knock-on effects for the stress levels of people’.* At a local level, a major reorganisation had taken place within the organisation in the period leading up to the trial, resulting in significant pressures on staff time and resources and low levels of trust between staff and senior management. The overall picture was one of significant job insecurity and uncertainty.

### Managerial pressures and stress

Whereas the educational intervention of the trial assumed senior managerial support (with the learning materials containing abstracted statements such as *‘Managers will be given the support they need to work with employees to help find proactive solutions to problems before they become too great’,* and *‘Make sure you are not stressed yourself …’*), the practical reality recounted by respondents was a rather different one. Managers described how they felt that their *‘hands were tied’*, and despite their best efforts, often felt powerless to effectively manage stress and help their employees. The unsupportive context within which managers felt they were working is illustrated in the narrative account in Table 2 below.

**Table 2:** A manager’s story of the stress of reorganisation

One (member of staff) who is very, let me just try and think of the wording for it, very committed, very conscientious, always increasingly finding it difficult because he couldn’t give his clients what they needed so that weighed heavy on him, couldn’t get his documentation up to date because that’s a demand from management that your documentation is up to date and trying to fulfil all those roles is really difficult. So we had supervision and it’s particularly since the change of this new redesign and what I tried to do with him is spend time out of the office so that he wasn’t part of things going on in the office so that he could quietly get on with some of his documentation in a quiet environment, tried to hold back any allocations for a bit until he felt more on top of his work … So I did listen and I did what I could but he could accept I was limited because the expectation on the team from higher management. I actually declared the service unsafe because the other stress on the team was that two (members of staff) went off on long-term sick and I realised I’d got to allocate all those cases because we haven’t got any (staff) that have light (caseloads) anymore and so that put extra strain and at that point I declared the service unsafe. … we keep on being told that there’s no money for extra staff. We ended up with a member of staff (from another team) coming over into our team and that was only because I fought for it because I said, ‘Look staff can’t continue under these levels’. But that person ended up resigning and he was brilliant, no job to go to, so I suppose using that as an example he was very positive about my input, he wasn’t positive about the higher management and … how they’ve managed this redesign and the pressure they’ve put on staff and he was very vocal about that on leaving. But I felt my hands were tied, I’d done as much as I could because I tried to support him through it … that came out on his exit interview and everything and when he resigned saying the job was untenable … (Manager, M1)	

Although the focus of the trial was on reducing employee stress, another finding to emerge from respondents’ accounts was the significance of managers’ own stress, and the difficulty of drawing a clear distinction between managerial and employee stress. Managers in the study were also employees, who talked extensively about the managerial practices of their senior managers, the lack of support they felt they were being given, the volume of work and lack of time to do it in, feeling they lacked control to manage workplace stress. They saw themselves as pushed in both directions, as *‘the damp proof course in the organisation, nothing permeates in either direction’* (Key Informant, KI2). Nevertheless, for the purposes of the trial methodology, participants had to be defined either as a manager or employee. Thus, here was another example of the tension between the demands of variables-based research and the reality of the social world. The trial necessarily focussed on managers as managers of employees and specifically of employee stress, but interviews with managers highlighted a more complex reality, and how this context was critical to understanding and interpreting the intervention.

Qualitative findings illuminated how, with the pressure on staff time and resources, managers struggled to find the time to engage with the intervention:

It was finding the time in the day just to sit down and be able to do it sat at my desk without some other priority or somebody knocking at my door with another question. That was really it, it wasn’t time consuming or anything necessarily it was just literally finding enough time … I found it was useful, it was something I would want … I didn’t get far enough through to really be able to say actually, ‘this could have been done differently …’ (Manager, M9)

### The intertwined nature of personal and workplace stress

A striking finding to emerge from manager and employee narratives of employee stress was how their accounts invariably started with a description of the employee as a family carer, of having an elderly mother, or a sick relative, or coping with a bereavement, or grandchildren who needed looking after whilst the parents were going through a messy divorce, or various other ‘private personal stresses’. As one respondent commented: *‘It’s not really work stress so much as it’s personal stress. But of course it does have an impact on one’s work life’* (Key Informant, KI13). The stories of stress at work that we present in this paper all convey the complex, intertwined nature of personal and workplace stresses.

Overall, our research highlighted that ‘context’ was not something that could be considered as a backcloth to the intervention and the trial, but rather was a dynamic, interacting part of organisational life, woven into the very fabric of what the trial was attempting to isolate and measure [35]. This ‘active’ and dynamic conceptualisation of context is a rather different one to that more commonly advanced in discussions of how qualitative research adds to RCTs by delineating a list of contextual factors that can somehow be captured and incorporated into a final trial design [36].

## Discussion

One conclusion that could be drawn from the results presented in this paper is that they unhelpfully complicate matters for those undertaking a trial. It could be argued that a trial design is necessarily and inevitably about simplifying the social world so that it can be studied through identifying and measuring variables and the statistical relationships between them. Qualitative research, on the other hand, is about exploring the complexity of social life, and based on a philosophical perspective (interpretivism) that rejects the idea that the world can be reduced to and represented by abstracted variables [8, 37]. Cohn et al. argue that acknowledging that the social world is complex ultimately requires that complexity is studied ‘*without fully unraveling it*’, and yet, an RCT design by its very nature requires *‘the isolation of measurable parts and hence the sacrifice of any genuine commitment to complexity’* [38]. Thus, it is clear that within mixed-methods research there are ever-present tensions between acknowledging complexity and simplification of the social world, between rich description and isolating and classifying elementary parts. In line with the pragmatist stance outlined above, we sought to engage with these tensions in a productive, integrative way [12].

There were many examples of the ways in which the GEM study team adopted this pragmatic position. From the outset, the principal investigator, research team and project steering committee welcomed the contribution they considered qualitative research could make to the trial. There was no sense in which the qualitative findings were seen as unhelpfully complicating matters; the qualitative study’s problematising and contextualising were embraced for the insights they offered. The qualitative accounts facilitated sense-making of the quantitative results, specifically helping to answer the question of *why* the intervention failed to have an effect on wellbeing, and this was made clear in the project’s final published report. The qualitative findings were seen as providing a wealth of learning about the possible factors influencing the likely effectiveness of the intervention, including: the problem of time, the lack of support from senior managers, the upheaval of organisational change, the specific characteristics of managers taking part, and the way in which they engaged (or not) with the intervention. Moreover, in reporting the conclusions of the study, the research team noted how the qualitative findings suggested ways in which the design of a full trial might be modified, for example, the need to include a measure of manager stress, the need for the trial to include a measure of the active learning elements of the educational intervention, and to do more to embed the intervention into organisational processes. Overall, the principal investigator reflected that: *‘without the qualitative, the final report would be quite a slim document and would not show much light on what was really going on’.* Thus, in certain respects the GEM study could be seen as an example of what O’Cathain et al. have referred to as the ‘integral-in-practice’ model of the relationship of qualitative research to the trial (in contrast to the ‘peripheral’, ‘add-on’ and ‘integral-in-theory’ models) [7].

This brings us back to the question posed in the Introduction of what does integration in practice mean? O’Cathain and colleagues defined the studies they reviewed as ‘integral-in-practice’ when the qualitative research was felt by the principal investigator to have had an impact on the trial by changing the outcome measures to be used or explaining the trial findings. In the GEM study the qualitative component certainly helped to explain the trial findings. However, it could be argued that a number of opportunities were missed for more in-depth integration and synthesis of the qualitative and quantitative research, leading us to label our study as ‘partially integrated’. We end this paper by exploring these opportunities, and discuss them within the context of recommendations from other mixed-methods researchers on maximising value of qualitative research to a trial.

Firstly, the GEM study proposal was developed without the input of a qualitative researcher. Whilst there was some leeway once the study was funded to develop the methodology of the qualitative component, the lack of expert qualitative input from the outset meant there was no opportunity for a qualitative perspective to feed into the fundamental study design. One helpful modification, for example, might have been to begin the study with some preliminary qualitative research to inform the design and help set the agenda for the quantitative work. Hesse-Biber and Johnson suggest that if mixed-methods research teams work together from the outset of a study they are then able to envision new ways of asking questions, and explore new ways to structure research to get at complex ideas [39].

Secondly, although the overall trial duration was 18 months, the proposal only applied for a 1-year appointment of a qualitative researcher (6 months into the start of the trial). This limited the role of the qualitative research in being able to document from the outset how the implementation process of the trial and intervention unfolded. Encouragingly, before the NIHR agreed to fund the study it requested that the proposal be modified to include some senior qualitative input to supervise the qualitative researcher. At this stage the senior qualitative researcher became a co-applicant. Other researchers have emphasised the importance of senior-level input to maximise the chance of high-quality qualitative design and integrative thinking [7]. Notwithstanding this modification, the GEM study was designed as a trial in which qualitative research was embedded, rather than a fully equal partner or, as in some cases, the trial embedded within the qualitative research [40]. Giddings has argued that, in the main, mixed-methods research studies are what she refers to as *‘positivism dressed in drag’* – in other words, the label of mixed-methods *‘covers for the hegemony of positivism’*, with qualitative research still very much the poor relation at the methodological level of overall thinking [41].

Thirdly, opportunities were missed for joint problematisation through more in-depth integrative team communication. Typically at research team meetings, the quantitative study and qualitative study were itemised as separate agenda items, and discussed as discrete endeavours, rather than time being explicitly built in for focussed team reflection on the implications of emerging qualitative findings for the trial. Although there were a few isolated examples of some integration at the level of data analysis (for example, the insights into managerial practice and contexts revealed by the qualitative findings prompted further exploratory quantitative analysis into how employees’ responses to primary and secondary outcomes differed according to whether they related to managers who were adherent or non-adherent to the intervention, which helped to give further understanding of why the intervention showed no overall effects), the research team failed to engage in more fundamental integrative thinking. Morgan recommends a ‘third effort’ phase of analysis (after analysis of the qualitative and quantitative components), noting that such a phase requires an explicit commitment of time and energy [42]. O’Cathain et al. describe three techniques that could help researchers to integrate qualitative and quantitative data in this ‘third phase’: triangulation (a process of systematically listing findings from each component of a study and exploring areas of convergence, complementarity, divergence and dissonance), following a thread (selecting a question or theme from one component and following it across the other component(s)), and a mixed-methods matrix (mapping all the data relating to single cases by identified themes) [43].

Within our study there was little explicit space for collective articulation of the impact of the qualitative research on the trial. It could be argued that ultimately the qualitative findings raised critical questions about the appropriateness of the experimental design of an RCT to study how an educational intervention for managers impacts on employee stress and wellbeing. The findings that learning is as much a social as an individual process, that there is no straightforward linear relationship between learning, knowledge and behaviour, the likely incompatibility between processes for maximising learning and the methodological requirements of a trial, the significance of tacit knowledge and practical judgement and the difficulties of measuring these human attributes within a variables-centred paradigm, perhaps all pointed to the need to consider an alternative to a trial design for the main study. However, during the study and final report writing-up period, the continuing assumption was that the full study would follow a similar trial design, albeit with some fine-tuning. It was only after receiving an editorial comment from the NIHR on the draft final report stating that *‘the review of the pilot is rather undermined by its apparent bias in terms of advocacy for a full trial, when what seems justified is further programme development and a broader adapted evaluation’* (personal communication feedback from the NIHR) that it was acknowledged that there needed to be *‘further mixed-method adaptation of the intervention in a further study rather than progression to a full trial’* (personal communication feedback to the NIHR). This resonates with a key recommendation of the NIHR HTA report on maximising value from qualitative research referred to at the beginning of this paper that mixed-methods research teams need to *‘design and implement studies not trials’* [1].

Finally, at the stage of publishing papers we divided the writing into the ‘quantitative’ and ‘qualitative’ papers, responding to the norms of publishing requirements and practices. O’Cathain and colleagues’ research have identified integration at the publishing stage as a common problem for mixed-methods research teams [7]. Bryman also notes how publication issues may hinder integration [44]. In the case of the GEM study the draft manuscript of the main study paper included very little about the qualitative study; encouragingly, however, journal reviewers requested that more detail be included, arguably indicating a growing awareness of the need for mixed-methods studies to be reported and published as such.

## Conclusion

Despite the growing popularity of mixed-methods studies and considerable emphasis on the potential value of qualitative research to the trial endeavour, there remains a dearth of published studies reporting on actual contribution. Our paper has provided a critically reflective account of our experiences as a mixed-methods team combining qualitative and quantitative research, and the opportunities for, and challenges of, integrative thinking. It has been suggested that the key to working with mixed-methods is for researchers to acknowledge the alternative conceptualisations of knowledge, and reflect on their position in relation to the range of possibilities throughout the research process [36]. Our own experience supports this view, although we found this something that was easier to do retrospectively, through the writing of the final report and this paper, than in real time, when the day-to-day demands of data collection and analysis for each component of our study perhaps inevitably took precedence over reflective thinking about integration. We recommend that future mixed-methods studies build in time for ongoing integrative thinking to ensure that qualitative and quantitative components add up to more than the sum of the parts.

## Abbreviations

GEM, Guided E-learning for Managers; HTA, Health Technology Assessment; NIHR, National Institute for Health Research; RCT, randomised controlled trial
